# Mechanisms Explaining the Longitudinal Effect of Psychosocial Safety Climate on Work Engagement and Emotional Exhaustion among Education and Healthcare Professionals during the COVID-19 Pandemic

**DOI:** 10.3390/ijerph21060698

**Published:** 2024-05-29

**Authors:** Kelly Bourgoin Boucher, Hans Ivers, Caroline Biron

**Affiliations:** 1 Department of Management, Faculty of Business & Administration, Université Laval, Québec, QC G1V 0A6, Canada; caroline.biron@fsa.ulaval.ca; 2 Center of Expertise for the Management of Occupational Health and Safety, Québec, QC G1V 0A6, Canada; 3 VITAM—Research Center for Sustainable Health, Université Laval, Québec, QC G1J 2G1, Canada; 4 School of Psychology, Université Laval, Québec, QC G1V 0A6, Canada; hans.ivers@psy.ulaval.ca

**Keywords:** psychosocial safety climate, job demands–resources theory, work engagement, emotional exhaustion, education professionals, healthcare professionals

## Abstract

During the COVID-19 pandemic, the education and healthcare sectors were severely affected. There is a need to investigate the ways in which these workers in at-risk sectors can be protected and through what mechanisms. The aims of this research are, therefore, (1) to assess the mediating role of job demands and resources in the relationship between psychosocial safety climate (PSC) and work engagement and emotional exhaustion, and (2) to test for sector-specific differences among education and healthcare professionals during the COVID-19 pandemic. In the study, which employed a longitudinal design including three measurement times, 70 education professionals and 69 healthcare professionals completed a questionnaire measuring PSC, psychological demands, social support, recognition, work engagement, and emotional exhaustion. The results show that PSC was significantly higher among education professionals than among healthcare professionals. When considering both job sectors together, mediation analyses show that social support mediates the PSC–work engagement relationship, while psychological demands mediate the PSC–emotional exhaustion relationship. Moderated mediation analyses show that job sector is a moderator: among education professionals, colleague support and recognition mediate the PSC–work engagement relationship, and psychological demands mediate the PSC–emotional exhaustion relationship. PSC is associated with more balanced job demands and resources, higher work engagement, and lower emotional exhaustion among education and healthcare professionals. The study of these two sectors, which are both vital to society but also more exposed to adverse work conditions, shows the importance that managers and executives must attach to their mental health by improving their respective working conditions.

## 1. Introduction

Several studies report a deterioration in psychological health since the start of the COVID-19 pandemic [1,2,3], with the education and health sectors being particularly vulnerable, even before the pandemic. In Quebec, schools were 5–18% understaffed, and the labour shortage was so severe during the pandemic that parents and unqualified staff had to be called in [4,5]. In Spain, during lockdown, primary and high school teachers have been reported to have higher levels of distress related to the workload in this context [6]. In Quebec’s healthcare sector, the shortage of nurses, respiratory therapists, and many other professionals created numerous problems, including service breakdowns and emergency room closures. For both sectors, the pandemic has been particularly disruptive in terms of working conditions, with many school closures, changing health measures, and various impacts on working conditions. In Japan, healthcare workers reported increased stress [7], while physicians and nurses in Brazil were feeling more tired [8]. During the second and third pandemic waves, half of the healthcare workers who participated in a study in Québec reported a high or very high level of psychological distress [9]. Of them, 82% perceived their psychological distress as work-related. Despite greater exposure to adverse working conditions, almost no study has considered the two sectors in conjunction and investigated whether the prevention mechanisms are the same.

Different studies have shown that psychosocial safety climate (PSC) helps to foster a healthier work environment and prevent psychological health problems [10,11]. The PSC represents employees’ shared perceptions of the organizational policies, practices, procedures, and systems that address employees’ psychological health [12]. The PSC is a precursor to the demands and resources that are essential for work engagement and to prevent emotional exhaustion [13]. Despite a significant number of studies on PSC, few have considered these two specific employment sectors during a time of crisis. This study aims to assess the mediating role of demands and resources in the PSC–work engagement and emotional exhaustion relationships, specifically among healthcare and education professionals, showing the differences between the two. A better understanding of the mechanisms in the psychosocial work environment by which PSC influences work engagement/emotional exhaustion can help to propose more specific avenues of prevention. In the next section, we first present the JD–R theory to highlight the demands and resources that are associated with emotional exhaustion and engagement of workers in the education sector and healthcare workers. Second, in Section 2.2, we describe the literature on psychosocial safety climate and its association with the JD–R in each of these sectors. Following the description of the theoretical models and their use in previous studies in these two sectors, we present the research hypotheses and objectives.

## 2. Literature Review and Theoretical Frameworks

### 2.1. Job Demands–Resources Theory

The role of resources is fundamental to job demands–resources theory (JD–R) [14]. Job characteristics can be classified into two distinct categories: job demands and job resources. Job demands are defined as the physical, psychological, social, or organizational aspects of the job that require sustained physical or psychological effort and which are associated with physical or psychological costs for the employee [15]. Job resources, on the other hand, are defined as physical, psychological, social, or organizational aspects of the job that are functional in achieving job goals, that reduce job demands and associated physical and psychological costs, or that stimulate personal growth, learning, and development. The demands and resources of employment thus give rise to two different processes—a health-impairment process and a motivational process [14]. On the one hand, the higher the demands, the more the psychological health will be negatively impacted, including increased emotional exhaustion. On the other hand, the more resources available, the higher the level of work engagement. These resources also play a role in psychological health, reducing the negative impact of demands.

During the pandemic, several studies reported increased job demands in many employment sectors [16,17,18]. The majority of these studies concentrated on the healthcare field, showing the extent to which the pandemic exacerbated existing high workload problems [19]. Among teaching staff, overload-related problems were also reported in several studies carried out before the pandemic [20,21]. Increased workload is a risk factor for the quality of psychological health [22,23]. During the pandemic, teachers had to adapt quickly to a changing context, with information delivered by the authorities at the very last minute. For example, on 13 March 2020, the Government of Quebec declared a health emergency and, 4 days later, announced that all teaching would have to take place remotely until 27 March [24]. Masks had to be worn for several months, in addition to the adoption of numerous disinfection measures, which may have aggravated workload problems. Teachers also had to devise distance learning methods for students and their parents [25]. Higher job demands also led to time management problems. Weaker time management among Canadian teachers was strongly associated with higher levels of burnout and cynicism [26]. Another study noted that the transition to full distance learning reduced teachers’ research and writing time, as well as the time they had available for students [27]. Among healthcare professionals, workload was higher for workers interacting with patients affected by COVID-19 [28]. Working long hours was associated with higher levels of stress, anxiety, and burnout [29]. Emotional demands (confrontation with death, human suffering, aggressive and troublesome patients) and quantitative demands (work intensity) were positively related to burnout and its three components of exhaustion, cynicism, and professional inefficacy [30]. However, only emotional demands were a predictor of burnout in all three regression models. Workload was also negatively related to work engagement [31]. Another study described the workload of healthcare staff as overwhelming during the early days of the pandemic, with high patient volumes and increasing numbers of severe cases of COVID-19 [32]. Employees also felt a loss of control in the face of the situation.

Several studies have shown that resources such as social support and recognition play a decisive role in both work engagement and burnout [33,34,35]. In addition to acting as a moderator to reduce the effect of demands on work engagement and burnout, resources help to foster engagement [14,36]. In a study among teachers and school counselors, perceived supervisor support was negatively related to stress levels [37]. Another study found a strong correlation between administrative support and burnout [26]. Similarly, the quality of their relations with superiors was one of the factors most associated with teachers’ wellbeing [38]. Among healthcare workers, social support was negatively correlated with various psychological health variables, namely, fear, stress [39], depression, and anxiety [39,40]. Another study found that peer support was primarily an important source of social support for healthcare workers, since colleagues experienced similar situations and were thus able to better understand their experiences [41].

A second resource that emerged as a determinant of wellbeing and burnout is recognition. In the education sector, a Finland study which used latent profiles identified three different profile groups of teachers: poorly engaged teachers with the highest effort and lowest reward (group 1), averagely engaged teachers with higher effort than reward (group 2), and highly engaged teachers with higher reward than effort (group 3) [42]. Results also suggest that teachers from the first group have significantly higher occupational stress than the two other groups. In the healthcare sector, effort and overengagement were positively related to depression and anxiety, while recognition was negatively related to depression and anxiety [43]. In another study, a large proportion of anesthetists had high levels of job stress and an imbalance between high effort and low recognition [44]. Effort was positively correlated with the presence of depressive symptoms. Furthermore, in one study, participants found that intrinsic rewards, such as recognition and gratitude, positively affected their psychological health [7].

Demands and resources are strongly influenced by practices, policies, and procedures related to psychological health [13]. Indeed, the PSC acts as a precursor, upstream of demands and resources.

### 2.2. Psychosocial Safety Climate

Closely associated with the JD–R model, PSC is a theory of workplace stress in which management prioritizes workers’ psychological health as much as productivity. It represents employees’ shared perceptions of the organizational policies, practices, procedures, and systems that address employees’ psychological health [12]. Four principles underpin the PSC: (a) the level of engagement and support from senior management towards stress prevention; (b) the priority that management gives to psychological health and safety versus productivity objectives; (c) two-way organizational communications with regard to psychological health and safety; and (d) the extent of participation and involvement of managers and employees with regard to psychological health and safety [45].

According to Dollard et al. [13], PSC is the cause of causes since it is a precursor to demands and resources. Several studies show that demands and resources mediate the relationship between PSC and burnout/work engagement [46,47,48,49,50]. PSC is said to be a multilevel theory because it influences job demands and resources, which in turn influence psychological health symptoms and work engagement/alienation, which in turn have an impact on health problems and work outcomes, and then on societal costs [45]. PSC is therefore a macro or contextual factor for the organization, acting on the psychosocial work environment; that is, on the conditions and organization of work. As numerous studies have shown, this psychosocial environment affects employees’ physical and psychological health [51,52,53,54].

PSC is negatively related to burnout and work demands, whether challenging or hindering [55]. These demands, on the other hand, are positively related to burnout and mediate the relationship between PSC and burnout. During the pandemic, “challenge” demands, which allow employees to increase their sense of personal accomplishment, and “hindrance” demands, which can reduce personal advancement and increase negative consequences such as anxiety and disengagement, were mediators of the relationship between PSC and burnout [56].

Before the pandemic, a low PSC score was found to be associated with high stress among healthcare workers [10], but social support failed to explain this relationship. During the pandemic, PSC was positively related to work engagement and negatively related to working compulsively [57]. PSC predicted a lower level of work engagement when working compulsively mediated their relationship. In another study, PSC predicted a lower level of presenteeism through its negative relationship with work intensification (an increased level of effort an employee needs to invest during the workday) [58].

Senior management’s commitment to stress prevention through practices, policies, and procedures has proved to be a determining factor in the mental health of staff during an abrupt, unplanned event such as COVID-19 [12]. In the study comparing two groups of work units during the pandemic—one that had undergone training, and the other that did not—PSC was higher in the work units that had undergone training, but significant improvements were made in organizational communication and management engagement in the other work units. Recent studies also suggest that the PSC and the commitment of managers and executives could be targeted directly in interventions to act upstream of psychosocial risks [12].

Several studies looking at the psychological health of teachers and healthcare workers during the COVID-19 pandemic used cross-sectional methods that only employed data collected at a single measurement time. There is, therefore, a need to enrich the literature with longitudinal studies. PSC has rarely been investigated in the context of the COVID-19 pandemic, and by contrasting two core, yet vulnerable, sectors, namely, the education and healthcare sectors. These two sectors are vital to the functioning of society but have been undermined (sometimes to the breaking point) by the pandemic and its many changes. To better prevent severe labour shortages in these two sectors, it is worth looking back to better understand what has affected staff in terms of work engagement and burnout, as well as their contributing mechanisms. These two sectors have also been treated differently in government policies. Among other things, healthcare staff have been referred to as “guardian angels” and offered various bonuses, but in the end many of these initiatives have been received cynically by the same staff [59]. In the education sector, less effort has been put into cushioning the impact of the pandemic. As highlighted by Beames et al. [60], the mental health of teachers has been a neglected research area despite their crucial role during the pandemic in maintaining children’s education and enabling parents to work. Differences in the mechanisms affecting work engagement and burnout between these two sectors remain largely unexplored. There are grounds for exploring differences in the processes that explain the effect of PSC on mental health. In a study among Malaysian teachers, Yulita et al. [61] showed that PSC was related to engagement through managerial support. In other words, teachers perceived that their organization cared about their psychological health via their managers’ practices (enacted PSC). Yet, Havermans et al. [10] found that a lower PSC was associated with higher stress in Dutch healthcare workers, but this association could not be explained by colleague support nor by supervisor support. As highlighted above, while a number of studies point to similarities between these two sectors, very few have considered them in conjunction and explored the possibility that the mechanisms by which the PSC affects their engagement and exhaustion are distinct.

**Hypothesis** **1.**  *There are differences between professionals in the education and healthcare sectors in terms of their perception of the psychosocial safety climate, their level of exposure to job demands and resources, and their levels of work engagement and emotional exhaustion.*

**Hypothesis** **2.**  *PSC at T1 is positively associated with job resources (social support, colleague support, superior support, and recognition) at T2, but negatively associated with psychological demands.*

**Hypothesis** **3.**  *Psychological demands at T2 are related to emotional exhaustion at T3, and resources (social support and recognition) at T2 are related to work engagement at T3.*

**Hypothesis** **4.**  *Psychological demands mediate the relationship between PSC and emotional exhaustion, and job resources mediate the relationship between PSC and work engagement.*

**Hypothesis** **5.**  *Job sector is a moderator of the relationship between PSC and work engagement and emotional exhaustion, mediated by job demands and resources.*

**Figure 1:**
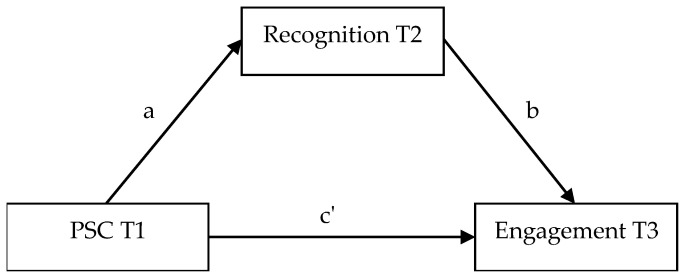
An example of one of the mediation models used (a refers to the “alpha” relationship, b to “beta” relationship and c′ to the indirect relationship).

This research aims to assess the mediating role of job demands and resources—namely, psychological demands, social support, colleague support, supervisor support, and recognition, in the relationships between PSC and work engagement and emotional exhaustion—and to ascertain differences by employment sector among education and healthcare professionals during the COVID-19 pandemic. First, we describe differences between education and healthcare professionals in their perceptions of their PSC, job demands, and resources, as well as their work engagement and emotional exhaustion during the pandemic. Second, we investigate the mediating effect of job demands and resources in the relationships between PSC and work engagement and emotional exhaustion (both sectors combined). Third, we assess whether employment sector (education vs. healthcare) is a moderator of the mediation patterns previously studied; that is, how each mediator’s contribution to the PSC–work engagement and emotional exhaustion relationships changes according to sector. Overall, the study aims to verify if PSC at T1 → job resources at T2 (social support, colleague support, superior support, and recognition) → engagement and emotional exhaustion at T3, and if there are differences in these relationships based on employment sector (see Figure 1 for an example of a mediation model).

## 3. Materials and Methods

### 3.1. Procedure

This longitudinal study was carried out over three measurement periods (April 2020, June 2020, and February 2021), and data collection was therefore distributed over almost the entire first year of the COVID-19 pandemic in Quebec. More specifically, data for the first measurement period were collected in April 2020, during the period of strict confinement required by Public Health, when nonessential services were closed, and education took place remotely. Data for the second measurement period were collected three months later at the end of June 2020, when stores, shops, and schools reopened. Data for the third measurement period were collected eight months later, in February 2021, after the introduction of a curfew and during the deconfinement plan [24]. The initial sample was collected using a web panel representative of the Quebec population. Web panels consist of a base of respondents who, if selected, commit to participate in future online surveys [62]. To encourage selected participants to take part in the survey, a random draw for a participation prize took place among the respondents at each measurement time. To encourage participants to return to complete the survey and to limit attrition, the value of the prize increased at each measurement time. The protocol was approved by Université Laval’s ethics committee.

### 3.2. Participants

The sample for this study was selected from a larger study on adult workers. A stratified (age, gender, geographical residence) random sample of 1450 eligible participants (that have worked in the last 7 days) was selected from a population-based web panel (N around 60,000) and agreed to take part in the survey. Since employment sector was not documented from the web panel, decision about final inclusion was postponed until the first measurement, where employment sector was reported by participants. From this sample, N = 183 respondents indicated they were workers from the education (n = 91) and healthcare (n = 92) sectors who identified as professionals (the choices being middle or senior management, first-level management, professional, administrative staff, technician, and blue-collar/trade). Since objectives are longitudinal in nature (this is not a time-lagged design), the selected participants for analyses also had to have completed the online survey at the first measurement time and at least two of the three measurement times used in the present study. Questionnaires were matched for each participant according to a coding key. The final sample for our study, therefore, consisted of N = 139 participants (76% of the original sample): 70 professionals from the education sector and 69 from the healthcare sector.

### 3.3. Measures

#### 3.3.1. Demographics Variables

The demographic variables chosen to describe the participants in our sample were gender, age, employment status, seniority, and employment sector. Employment sector classification was based on the North American Industry Classification System [63], which was previously used in the Enquête québécoise sur des conditions de travail, d’emploi et de SST (EQCOTESST) [64].

#### 3.3.2. Psychosocial Safety Climate

The PSC was measured at the first measurement time (T1) with the PSC-4, a 4-item measurement scale that takes into account respondents’ perceptions of the priority given to psychological health problems by their organization’s top management [65]. Since a validated French-Canadian version of the questionnaire was not available, a backward translation was made [66]. The scale responses ranged from 1 (strongly disagree) to 5 (strongly agree), and the sum of individual items was used. An example item was “There is good communication here about psychological safety issues which affect me”. This variable was measured at the first measurement time, and the internal validity of the scale is high (α = 0.922).

#### 3.3.3. Mechanisms: Job Demands and Resources

Job demands and resources (psychological demands, social support, and recognition) were measured at the second measurement time (T2). The scales used to measure these mechanisms are the same as those included in the EQCOTESST [64]. The psychological demands and social support subscales were adapted from the short version of the Job Content Questionnaire (with permission) [67], whose French translation was found to have acceptable psychometric properties [68,69], and the recognition subscale was adapted from the Effort–Reward Imbalance Questionnaire (ERI) by Siegrist [70]. For these measures, responses on the various scales ranged from 1 (strongly disagree) to 4 (strongly agree). When an item presented a negative framing, its answer was reverse-coded to match other items.

##### Psychological Demands

Psychological demands were measured using six items measuring the extent to which participants had to work fast, work excessively, face conflicting demands, and be interrupted in their tasks. This variable was measured at the second measurement time, where the internal consistency of the scale was high (α = 0.795).

##### Social Support

Social support at work was measured by considering the support of colleagues (three items) and to what extent participants perceive that they are part of a team, that their colleagues facilitate the execution of the work, or are hostile (reversed item). Four items were used to assess whether participants perceive their superior as helpful, paying attention, competent in getting people to work, and hostile (reversed item). Social support (overall) was measured using all seven items. Internal consistency of the scale was generally high (α = 0.674 for colleague support, and α = 0.809 for superior support, α = 0.825 for social support overall).

##### Recognition

The recognition scale was adapted from the Effort–Reward Imbalance Questionnaire (ERI) [70]. The French translation has been validated for its psychometric properties [71], and eight items were derived from this scale. These covered various forms of rewards (esteem from colleagues and superior, fairness, promotion opportunities, and adequate salary). An example item was “Given all my efforts and achievements, I receive the respect and esteem I deserve at work”. This variable was measured at the second measurement time, and the internal consistency of the scale was acceptable (α = 0.698).

#### 3.3.4. Work Engagement

The scale used to measure work engagement at the third measurement time (T3) was the ultra-short version of the Utrecht Work Engagement Scale (UWES-3) [72], which has previously been tested for internal validity. The scale responses ranged from 1 (strongly disagree) to 5 (strongly agree). An example item was “I’m bursting with energy for my work”. The internal consistency of the scale was acceptable (α = 0.721).

#### 3.3.5. Emotional Exhaustion

Emotional exhaustion was measured at T3 using the corresponding subscale from the Maslach Burnout Inventory-General Survey (MBI-GS) [73]. The scale responses ranged from 1 (never) to 7 (all the time). An example item was “I feel tired when I get up in the morning and have to face a new day at work”. The internal consistency of the scale was high (α = 0.917).

### 3.4. Statistical Analyses

Descriptive sample analyses and correlations between the variables under study were performed using SPSS software version 27. The alpha level was set at 5% two-tailed by default. Mediation analyses of the relationship between PSC and work engagement and emotional exhaustion were performed using the coefficient product approach [74] using Mplus 7.0 software [75]. Specifically, two relationships were estimated: the “alpha” relationship (between the independent variable and the mediator) and the “beta” relationship (between the mediator and the dependent variable). The significance of the indirect relationship (calculated as the product of these two regression coefficients) was established by checking the confidence interval, calculated using a bootstrap approach (N = 5000) [76]. Subsequently, using the constraint functionality approach available in Mplus, a moderation test of the indirect relationship [77] was carried out by comparing the estimated indirect relationship in two sectors (education and health) and then calculating the confidence interval around this difference by resampling. Given the population-based nature of the survey, a sampling weight was applied for all analyses to ensure that the sample was representative of the population surveyed (people employed in Quebec in 2020–2021).

## 4. Results

### 4.1. Socio-Demographic Characteristics of Participants

Table 1 shows the sociodemographic characteristics at the first measurement time for each employment sector studied and for the total sample. The employment sectors grouped together a similar number of participants, and were statistically similar in terms of age, gender, and seniority within the organization. The total study sample consisted of 139 professionals from the education and healthcare sectors, 78.42% of whom were women (21.58% men), with an average age of 44.4 years. There were 70 education professionals, 71.43% of them women (28.57% men), with an average age of 46.03. Of these, 65.71% were part-timers, with an average seniority of 14.45 years in their current organization. There were 69 healthcare professionals, 85.51% of whom were women (14.49% men), with an average age of 42.75 years. Of these, 73.91% were full-time workers, with an average seniority of 10.01 years in their current organization.

### 4.2. Correlation Matrix

Table 2 shows the correlations between the variables. Using G*Power 3.1.9.7 software [78] and standard power conditions (1 − β = 80%, alpha = 5%, one/two-tailed test), correlations larger than ±0.29/0.33 could be detected with an expected sample size of n = 70 (since analyses were performed by domain, not overall). Over 50% of the correlations from Table 2 have a coefficient larger than 0.30.

### 4.3. Statistical Comparison of Descriptive Variables by Employment Sector

The first objective was to describe the differences between healthcare and education professionals in terms of their perception of their PSC and their demands and resources, as well as their work engagement and emotional exhaustion during the pandemic. Difference tests (Student’s *t*-tests) were performed on all descriptive variables used in the analyses to determine whether there were any differences between education and healthcare professionals. Our first hypothesis—that there are differences between professionals in the education and healthcare sectors in terms of their perception of the PSC, their level of exposure to job demands and resources, and their levels of work engagement and emotional exhaustion—was not supported, except for PSC (Table 3). PSC was significantly higher in the education sector than in the healthcare sector (*p* = 0.015). However, there was no significant difference for the other variables studied.

### 4.4. Mechanisms Explaining the Relationship between PSC and Employees’ Emotional Exhaustion and Work Engagement

The second objective was to investigate, by considering the overall sample, the mediating effect of job demands and resources in the relationship between the PSC and emotional exhaustion and work engagement. As mentioned before, mediation analyses of the relationship between PSC and work engagement and emotional exhaustion were performed using the bootstrap coefficient product approach [74]. As shown in Table 4, when engagement was used as DV, PSC at T1 predicted lower job demands at T2, and higher social support and recognition at T2. When emotional exhaustion was used as DV at T3, PSC at T1 predicted lower job demands, higher social support (overall) and social support from colleagues, and higher recognition at T2. Hypothesis 2 was, thus, mostly supported. Hypothesis 3 aimed to test beta relationships between the mediators and the DVs. Results in Table 4 show that colleague and superior support, as well as overall support at T2, promoted work engagement at T3. Job demands at T2 were associated with increased exhaustion at T3. Superior support at T2 was associated with reduced exhaustion at T3. Hypothesis 3 was, thus, partially supported.

Hypothesis 4 looked at indirect relationships and stated that psychological demands were a mediator of the PSC–emotional exhaustion relationship, and that job resources—social support, colleague support, superior support, and recognition—were mediators of the PSC–work engagement relationship. This was partially validated. The results showed that the indirect relationship of only one model was significant (*p* = 0.042), that of the relationship between PSC and work engagement, when social support was the mediator. In addition, one model was marginally significant when psychological demands (*p* = 0.089) was a mediator of the relationship between PSC and emotional exhaustion. In the two models above, both the alpha, the relationship between the independent variable and the mediator, and beta, the relationship between the mediator and the dependent variable, were significant. Detailed results are shown in Table 4.

### 4.5. Job Sector as a Moderator

The third objective was to assess whether sector of employment was a moderator of the mediation patterns previously studied; that is, how each mediator’s contribution to the PSC–work engagement and emotional exhaustion relationships changes according to sector. A moderation test of the indirect relationship was carried out by comparing the estimated indirect relationship in two sectors (education and health) and then calculating the confidence interval around this difference by resampling [77]. Hypothesis 5 stated that the sector of employment was a moderator of the PSC–work engagement and emotional exhaustion relationships. To establish whether the indirect relationship differed by sector (moderated mediation), the previous mediation models were therefore repeated by sector. The results showed that the indirect relationship between PSC and work engagement, when colleague support was a mediator, was significant among professionals in the education sector. In this model, the beta relationship—that is, the relationship between colleague support and work engagement—was significant. However, the alpha relationship—that is, the relationship between PSC and colleague support—was not significant. In addition, there was no significant difference in mediation between the two employment sectors. The model of the relationship between PSC and emotional exhaustion, when psychological demands were the mediator, was marginally significant for education professionals, with the lower bound of the confidence interval being a value of 0.001. In this model, the alpha and beta relationships were not significant in either job sector nor was there any significant difference in the indirect relationship between the two employment sectors. The model of the relationship between PSC and work engagement, where recognition was the mediator, was marginally significant for education professionals, with the lower bound of the confidence interval at −0.001. In this model, the alpha relationship, or the relationship between PSC and recognition, was significant for both employment sectors. The beta relationship, or the relationship between recognition and work engagement, was not significant. The difference between the two employment sectors was marginally significant, with the lower bound of the confidence interval at −0.001. The results of moderated mediation tests are presented in Table 5.

## 5. Discussion

This study took place in the context of the COVID-19 pandemic, which has severely deteriorated general psychological health in the population [1,2,3], and, in particular, certain employment sectors that have been particularly affected by problems of absenteeism and labour shortages. The aim of this longitudinal study was to assess the mediating role of job demands and resources in the relationship between PSC and work engagement and emotional exhaustion among education and healthcare professionals in Quebec during the COVID-19 pandemic, and to test for differences between the two employment sectors. Our results showed that PSC was significantly different between the two employment sectors under study. Professionals in the education sector report a higher level of PSC than those in the health sector (13.68 vs. 11.80). In Sweden, Berthelsen et al. [80] conducted a study to establish PSC benchmarks using organizational compliance with Occupational Safety and Health (OSH) regulations as a criterion. A high PSC score (>12) indicates “good” OHS practices, a medium PSC score (8–12) indicates “fair” practices, while a low PSC (<8) corresponds to “poor” OHS practices. Using Swedish benchmarks, we can see that the PSC in the education sector was considered as “high” and would correspond to good OHS practices, while the PSC of the healthcare sector would suggest a greater risk of noncompliance with current OHS regulations. Such benchmarks are being developed in Quebec but are not yet available.

As several previous studies have shown, PSC at T1 was positively associated with resources at T2, such as social support and recognition [46,81]. PSC was also associated with lower job demands. Social support at T2 was positively related to work engagement at T3, and superior support at T2 was negatively related to emotional exhaustion at T3. On the other hand, greater psychological demands at T2 are associated with greater emotional exhaustion at T3. Also, only social support acts as a mediator in the relationship between PSC and work engagement when considering both job sectors together, which means that social support is a mechanism by which PSC promotes work engagement. As for the moderating role of employment sector, colleague support (significant) and recognition (marginally significant) mediated the PSC–work engagement relationship, while psychological demands (marginally significant) mediated the PSC–emotional exhaustion relationship, but only among education professionals. However, the difference between the two job sectors is only marginally significant when recognition mediates the PSC–work engagement relationship.

### 5.1. Sector Differences

Hypothesis 1 was that there were differences in means between sectors for the variables under study. Only one significant difference emerged from our analyses, and that was in the perceptions of PSC. Education professionals showed a significantly higher level of PSC than healthcare professionals. In other words, it appears that senior management in the healthcare sector is perceived as giving a lower priority to the psychological health of its employees than senior management in the education sector. We hypothesized that employment status has influenced the perception of the PSC, considering that our sample comprises a large proportion of part-time workers from the education sector, whereas most healthcare professionals were full-time workers. Considering that data were collected at the beginning of the pandemic when schools were just reopening after being closed for six weeks, education professionals could have had a more positive view of PSC because they were working fewer hours, whereas healthcare workers were exposed to several psychosocial constraints, including higher job demands, ethical conflicts, and shortages of staff and equipment, all of which were detrimental to their psychological health [82]. However, further analyses did not reveal any differences according to employment status in either sector. The other variables studied, including emotional exhaustion, have similar averages across employment sectors. Very few studies compare these two employment sectors specifically. In partial contrast to our findings, a prepandemic study of teachers and nurses in Jordan showed that burnout is significantly higher among nurses [83]. Likewise, another study in Nigeria arrived at similar results; namely, that nursing staff display higher levels of burnout than teachers [84]. The disparity between these results and ours may stem from the context of our study. Given the abrupt onset of the COVID-19 pandemic and its impact on workplaces, professionals in both employment sectors had to palliate many changes in a short period of time. These two groups may well have experienced this period in a similar way, hence the similarity in all other descriptive variables, except for PSC.

### 5.2. Job Demands and Resources as Mediators

Our Hypothesis 2 is that the PSC was negatively related with psychological demands but positively related with social support, colleague support, superior support, and recognition. This hypothesis was partially validated by the results obtained for alpha relations in the mediation analyses. When PSC at T1 is high, psychological demands at T2 are lower, while recognition, social support, and colleague support at T2 are higher. A high level of PSC was already linked to lower job demands in a sample of university teachers before the pandemic [55]. Several other studies show the decisive role of PSC in predicting a higher level of resources and a lower level of demands [16,46,48]. The present study corroborates these findings, this time with a longitudinal design and participants from two specific employment sectors that were particularly affected by the upheavals brought about by the pandemic. However, in contrast to the study by Idris et al. [48], PSC at T1 has no significant relation with superior support at T2. It is possible that the COVID-19 pandemic and the stress generated by its suddenness impacted superiors’ attitudes towards their employees. Superiors may, therefore, have adopted an attitude that was inconsistent with the PSC perceived by professionals in both sectors, given that they themselves were trying to adjust to and endure the new situation. Indeed, as previously demonstrated, managers are not immune to burnout and a decline in the quality of their managerial work [11]. Also, it is possible that the government issuing contradictory directives over time, through the centralization of decision making, could have led superiors to have ambiguous relationships with their employees and, therefore, to be unable to support them like before the pandemic.

The results of the beta relationships partially validated Hypothesis 3. First, a high level of psychological demands at T2 was associated with a high level of emotional exhaustion at T3, which is consistent with other studies. The literature is no different in this respect. Studies during the pandemic showed that workload and long working hours were positively related to burnout [29,85]. Next, social support, colleague support, and superior support at T2 were positively associated with work engagement at T3, while superior support was negatively associated with emotional exhaustion at T3. In addition, administrative support was negatively correlated with teacher burnout [26]. Before the pandemic, work engagement was already linked to supervisor support and social support among healthcare staff [86,87] and university teachers [88]. Furthermore, recognition at T2 was not associated with work engagement at T3. This contrasts with the current scientific literature, where rewards predict a higher level of engagement [89,90] and an effort–reward imbalance predicts lower levels of engagement [91]. It is possible that the arduous nature of the work and the difficult working conditions in a pandemic context have had a detrimental effect on work engagement beyond what recognition can generally offset.

Our Hypothesis 4 is that psychological demands mediate the PSC–emotional exhaustion relationship, and that job resources mediate the PSC–work engagement relationship. This was only partially validated by two of the ten mediation models. Psychological demands mediate the PSC–emotional exhaustion relationship, while social support mediates the PSC–work engagement relationship. As these mechanisms have already been shown to mediate the health erosion and motivational processes initiated by PSC [16,48,50], they still have a role to play during the pandemic.

### 5.3. Employment Sector as Moderator

Finally, our Hypothesis 5 is that employment sector is a moderator of PSC–work engagement and emotional exhaustion relationships, mediated by job demands and resources. It could not be supported as the employment sector was only marginally significant when psychological demands mediated the PSC–emotional exhaustion relationship. Colleague support (significant) and recognition (marginally significant) mediated the PSC–work engagement relationship only for teachers, but the difference between sectors was not significant. While these two resources did not stand out in our mediation models across all sectors, they were mechanisms mediating the PSC–work engagement relationship, but only among teachers. The difference between the two sectors is marginally significant for the recognition model. As mentioned previously, recognition does not mediate the PSC–work engagement relationship when the two employment sectors are combined. In both sectors, PSC is significantly associated with recognition. It is likely that professionals from the healthcare sector no longer relied on recognition to stay engaged at work. For example, healthcare staff have been referred to as “guardian angels” and offered various bonuses, but recognition varied by profession and there were feelings of unfairness and inequitable treatment [59]. In addition, delays in payment of COVID-19 premiums and salary adjustments also add to the context [92]. It seems that recognition in this sector was not conducive to greater work engagement.

### 5.4. Contributions and Implications

The contributions of this study are multiple. First, in terms of theoretical contribution, the present study corroborates several others that show the importance of PSC and job demands and resources on work engagement and emotional exhaustion [93,94]. However, our study was conducted in the COVID-19 pandemic context. Furthermore, our study is longitudinal, allowing us to assess the extent of the relationships between PSC and work engagement and emotional exhaustion through the job demands and resources used as mediators among education and healthcare professionals. Indeed, few studies used a longitudinal methodology during the crisis period; most used a cross-sectional methodology instead, which only gives a snapshot of a specific point in time. The present study shows that the PSC is related to job demands and resources, and that these are associated with emotional exhaustion (particularly psychological demands) and work engagement (particularly social support). There are also few studies using the PSC during this same period, and those using it with education or healthcare staff are very rare. Moreover, to the best of our knowledge, no studies compared these two vulnerable sectors—the education sector and the healthcare sector—in terms of their psychological health during the COVID-19 pandemic.

Our results have practical implications for managers in these two sectors. Like many other studies [13], this study shows that the CSP is an important determinant of working conditions and, in turn, of psychological health. However, our study shows that PSC was lower in the *healthcare sector*. Training should be offered to *managers* and *top management* in the healthcare sector so that they can integrate psychological health as a value in their organization. Indeed, a recent study suggests that training middle managers increases the level of PSC within four months [12]. Considering the recent and ongoing strikes in the Quebec healthcare sector, where employees and their unions were claiming better working conditions [95], and PSC being a precursor to job demands and resources [13], PSC training would be beneficial for *healthcare managers* and *employees*, but also for *society* as a whole, since citizens are on the receiving end of treatments and better working conditions would naturally lead to higher quality treatments.

Also, recognition is a mediator of the PSC–work engagement relationship among *education professionals* (marginally significant). In the motivational process, a high level of work engagement is associated with greater job satisfaction [14]. Recognition is, therefore, a powerful lever for stimulating engagement in the education sector. In November and December 2023, several unions in the education sector went on strike with a number of demands, one of the main issues being recognition at work [96]. Students affected by these strikes, led by two different major groups, missed around ten days of school for some, and five weeks for others [97]. The education sector’s heavy-handed decision to go on strike, despite the consequences for students, shows that the *government* and *society* would benefit greatly from listening to the needs of the education sector’s employees and from better recognizing their work and the considerable efforts they make to establish a healthy balance. We recommend that *governments* and *senior executives* show greater benevolence and give higher priority to the psychological health of staff in these two sectors, when drawing up public policies, given the crucial role of healthcare and education for our societies.

Additionally, for both sectors, psychological demands were a mediator of the PSC–emotional exhaustion relationship (marginally significant), while social support was a mediator of the PSC–work engagement relationship. A high level of psychological demands was significantly associated with a high level of emotional exhaustion, and a high level of social support was associated with a high level of work engagement. Therefore, we believe it would be important for *managers* to use management practices that support workers’ psychological demands and social support. For example, *managers* and *employees* could sit together to review job descriptions and ensure that they are accurate and well balanced. Also, communities of practice could be set up to enable *employees* to develop their competencies, lessening the psychological demands’ effects on their wellbeing, while also empowering their interpersonal relations, creating a better sense of social support received. In fact, in a study, an online professional community of practice has helped develop social relationships among its members and obtain support through their social network [98].

### 5.5. Limitations

To properly interpret the results of our study, we need to consider its limitations. Although the response rate might seem low, it is not unusual for a web panel [99], especially since some of the participants among the 6000 invited were not included as they were unemployed over the week preceding the study. Another limitation of our study lies in the heterogeneity of the sample. The two categories of professionals used in our study include more than one body of work within each, making it difficult to target specific professionals such as elementary school teachers or nurses. The size of our overall sample (n = 139) is relatively small and did not allow for larger model analyses. Consequently, we had to proceed with three-variable mediation models: an independent variable, a mediator, and a dependent variable. These models, therefore, make it impossible to compare the variance explained by each of the job demands and resources when put together in the same model. The large number of tests conducted can increase the probability of Type I errors. We also present marginally significant results alongside our significant results, which could be slightly problematic. Despite these limitations, the sample selected was drawn from a panel representative of the Quebec population and enabled us to test longitudinal relationships between variables. The original sample was representative of the Quebec population in terms of age, gender, and geographical distribution. However, as we selected a subsample of health and education professionals, the sample used for this study is not representative of professionals in these two sectors. Nevertheless, compared with other studies using convenience samples, our sample is drawn from a representative population sample.

## 6. Conclusions

This longitudinal study took place against the backdrop of the COVID-19 pandemic, a succession of dynamic events which severely deteriorated the psychological health of the general population, and in particular the psychological health of certain employees that were particularly affected by absenteeism and labour shortages. Our aim was to assess the mediating role of mechanisms, such as job demands and resources, in the relationship between PSC and engagement and burnout among education and healthcare professionals in Quebec during the COVID-19 pandemic, and then to ascertain whether there were any differences between the two employment sectors. The results showed that the PSC was higher among education professionals than healthcare professionals during the pandemic. The results also enabled us to show that job demands and resources are mechanisms acting on the PSC–work engagement and emotional exhaustion relationships.

Future PSC research involving comparing employment sectors should be conducted using a larger sample. In addition, it would be interesting to use more specifically targeted professionals, such as teachers and nurses, since the current study includes these professionals in more than one job category. In addition to including other job demands and resources, future research could also target other emerging risks, such as ethical conflicts [82]—including moral injury and a lack of personal protective equipment among nursing staff—and short notice periods among teachers. Technostress is another emerging risk that is increasingly affecting these employment bodies, including teachers [100]. For example, during the pandemic, teachers had to work remotely, often with a lack of knowledge, skills, and resources for distance learning [101]. While some of these emerging risks might not be fully relevant in studies conducted in the post-pandemic context, they may have led to long-term consequences. Understanding these emerging risks could also be beneficial to better manage future crises.

## Figures and Tables

**Table 1:** Sociodemographic characteristics of participants.

	Education Professionals n = 70	Healthcare Professionals n = 69	All Combined n = 139
Gender			
Woman	50 (71.43%)	59 (85.51%)	109 (78.42%)
Man	20 (28.57%)	10 (14.49%)	30 (21.58%)
Age			
Mean	46.03	42.75	44.4
Standard deviation	9.34	10.88	10.23
Range	27–69	23–70	23–70
Employment status			
Full-time	24 (34.29%)	51 (73.91%)	75 (53.96%)
Part-time	46 (65.71%)	18 (26.09%)	64 (46.04%)
Seniority			
Mean	14.45	10.01	12.25
Standard deviation	9.08	9.137	9.35
Range	0–35	0–41	0–41

**Table 2:** Correlation matrix.

		PSC T2	Psy. Dem. T2	Col. Sup. T2	Sup. Sup. T2	Soc. Sup. T2	Rec. T2	PSC T3	Work Eng. T3	Emo. Exh. T3
PSC T1	E	0.61 ***	−0.22 Ϯ	0.25 *	0.35 **	0.32 **	0.32 **	0.61 ***	0.27 *	−0.21 Ϯ
	H	0.49 ***	−0.28 *	0.26 Ϯ	0.22	0.31 *	0.47 ***	0.66 ***	0.19	−0.05
PSC T2	E		−0.45 ***	0.17	0.42 ***	0.30 **	0.62 ***	0.70 ***	0.26 *	−0.19
	H		−0.33 **	0.23 *	0.56 ***	0.48 ***	0.50 ***	0.72 ***	0.38 ***	−0.07
Psychological demands T2	E			−0.12	−0.35 **	−0.29 *	−0.53 ***	−0.47 ***	−0.17	0.44 ***
	H			−0.04	−0.39 **	−0.38 **	−0.58 ***	−0.35 **	0.11	0.22
Colleague support T2	E				0.57 ***	0.88 ***	0.29 *	0.15	0.26 Ϯ	−0.05
	H				0.29 *	0.83 ***	0.17	0.27 *	0.07	−0.01
Superior support T2	E					0.86 ***	0.61 ***	0.31 **	0.07	−0.10
	H					0.79 ***	0.33 **	0.23 *	0.30 *	−0.03
Social support T2	E						0.36 **	0.23 *	0.13	−0.09
	H						0.29 *	0.30 **	0.23 *	−0.07
Recognition T2	E							0.45 ***	0.27 *	−0.30 **
	H							0.50 ***	0.10	−0.06
PSC T3	E								0.23 *	−0.31 **
	H								0.35 **	−0.20 Ϯ
Work engagement T3	E									−0.33 **
	H									−0.47 ***

Note. E = education sector, H = healthcare sector. Ϯ *p* < 0.10, * *p* < 0.05, ** *p* < 0.01, *** *p* < 0.001.

**Table 3:** Statistical comparison for descriptive variables by employment sector (Student’s *t*-tests).

Variable	Education M (SD)	Healthcare M (SD)	*t* (DF)	Cohen’s d
PSC T1	13.68 (3.72)	11.80 (4.92)	2.46 * (118)	0.43
Psychological demands T2	2.56 (0.57)	2.49 (0.56)	0.58 (85)	0.13
Colleague support T2	3.19 (0.48)	3.26 (0.58)	−0.54 (84)	−0.12
Superior support T2	3.08 (0.42)	3.18 (0.51)	−0.91 (83)	−0.20
Social support T2	10.76 (1.37)	11.11 (1.66)	−1.05 (85)	−0.23
Recognition T2	2.76 (0.41)	2.81 (0.38)	−0.57 (85)	−0.12
Work engagement T3	3.43 (0.58)	3.66 (0.76)	−1.50 (83)	−0.33
Emotional exhaustion T3	2.96 (1.40)	3.08 (1.31)	−0.41 (83)	−0.09

* *p* < 0.05, d = 0.20 (small effect), 0.50 (moderate effect), 0.80 (large effect) [79].

**Table 4:** Contribution (nonstandardized) of the six mediators in the PSC–work engagement and emotional exhaustion relationships (mediation analyses).

	Work Engagement			Emotional Exhaustion		
Mediator	PSC→Med (α)	Med→DV (β)	Indirect Relation (α × β)	PSC→Med (α)	Med→VD (β)	Indirect Relation (α × β)
Psychological demands	−0.183 **	0.147	−0.027	−0.170 *	0.454 *	−0.077 Ϯ
Social support	0.436 *	0.110 **	0.048 *	0.470 *	−0.050	−0.023
Colleague support	0.122 Ϯ	0.344 *	0.042	0.139 *	0.064	0.009
Superior support	0.045	0.402 ***	0.018	0.061	−0.625 *	−0.038
Recognition	0.155 ***	0.032	0.005	0.155 ***	−0.304	−0.047

Ϯ *p* < 0.10, * *p* < 0.05, ** *p* < 0.01, *** *p* < 0.001.

**Table 5:** Difference in the (nonstandardized) contribution of the six mediators in the PSC–work engagement and emotional exhaustion relationships, by sector (moderated mediation analyses).

		Education		Healthcare		Indirect Relation (IC 95%)		
Med	DV	PSC→Med	Med→DV	PSC→Med	Med→DV	Education (a)	Healthcare (b)	Difference (a − b)
Psychological demands	Work engagement	−0.278	0.101	−0.299	0.212	−0.018 (−0.125–0.023)	−0.039 (−0.174–0.024)	0.022 (−0.085–0.157)
Psychological demands	Emotional exhaustion	−0.260	0.261	−0.287	0.177	−0.101 Ϯ (−0.295–0.001)	−0.052 (−0.285–0.034)	−0.049 (−0.260–0.148)
Social support	Work engagement	0.318 *	0.211	0.313	0.256	0.042 (−0.012–0.131)	0.050 (−0.010–0.165)	−0.008 (−0.115–0.103)
Social support	Emotional exhaustion	0.334 *	−0.103	0.335	0.017	−0.051 (−0.209–0.055)	0.006 (−0.308–0.172)	−0.057 (−0.293–0.226)
Colleague support	Work engagement	0.258	0.483 *	0.181	0.127	0.078 * (0.006–0.180)	0.014 (−0.019–0.140)	0.064 (−0.036–0.178)
Colleague support	Emotional exhaustion	0.308 *	−0.010	0.190	0.087	−0.005 (−0.133–0.197)	0.017 (−0.065–0.245)	−0.022 (−0.249–0.192)
Superior support	Work engagement	0.114	0.443 *	0.079	0.237	0.032 (−0.043–0.108)	0.012 (−0.045–0.086)	0.020 (−0.077–0.121)
Superior support	Emotional exhaustion	0.152	−0.318	0.103	−0.165	−0.072 (−0.223–0.040)	−0.017 (−0.280–0.056)	−0.055 (−0.246–0.146)
Recognition	Work engagement	0.251 *	0.250	0.441 *	−0.264	0.039 Ϯ (−0.001–0.132)	−0.073 (−0.248–0.017)	0.112 Ϯ (−0.001–0.285)
Recognition	Emotional exhaustion	0.271 *	−0.155	0.426 *	0.019	−0.063 (−0.245–0.012)	0.008 (−0.259–0.219)	−0.071 (−0.330–0.215)

Ϯ *p* < 0.10, * *p* < 0.05.

## Data Availability

The raw data supporting the conclusions of this article will be made available by the authors on request.
